# Genetic Variants in Oxidative Stress-Related Genes and Their Impact on Prognosis and Treatment Response in Chronic Myeloid Leukemia Patients

**DOI:** 10.3390/ijms26125682

**Published:** 2025-06-13

**Authors:** Raquel Alves, Filipa Ventura, Joana Jorge, Gilberto Marques, Margarida Coucelo, Joana Diamond, Bárbara Oliveiros, Amélia Pereira, Paulo Freitas-Tavares, António M. Almeida, Ana Cristina Gonçalves, Ana Bela Sarmento-Ribeiro

**Affiliations:** 1 Laboratory of Oncobiology and Hematology (LOH) and University Clinics of Hematology and Oncology, Faculty of Medicine University of Coimbra (FMUC), University of Coimbra, 3000-548 Coimbra, Portugal; 2 Coimbra Institute for Clinical and Biomedical Research (iCBR)—Group of Environmental Genetics of Oncobiology (CIMAGO), Faculty of Medicine University of Coimbra (FMUC), University of Coimbra, 3000-548 Coimbra, Portugal; 3 Center for Innovative Biomedicine and Biotechnology (CIBB), 3004-504 Coimbra, Portugal; 4 Clinical Pathology Service, Centro Hospitalar Universitário de Coimbra (CHUC), 3000-061 Coimbra, Portugal; 5 Hematology Service, Centro Hospitalar Universitário de Coimbra (CHUC), 3000-061 Coimbra, Portugal; 6 Hemato-Oncology Laboratory, Instituto Português de Oncologia de Lisboa Francisco Gentil EPE, 1099-023 Lisbon, Portugal; 7 Laboratory of Biostatistics and Medical Informatics, Faculty of Medicine University of Coimbra (FMUC), University of Coimbra, 3000-548 Coimbra, Portugal; 8 Medicine Service, Hospital da Luz, 3020-479 Coimbra, Portugal; 9 Medicine Department, Hospital Distrital da Figueira da Foz, EPE, 3094-001 Figueira da Foz, Portugal; 10 Orthopedic Oncology Department, Centro Hospitalar Universitário de Coimbra (CHUC), 3000-061 Coimbra, Portugal; 11 Hospital da Luz Lisboa, 1500-650 Lisbon, Portugal; 12 Centro de Investigação Interdisciplinar em Saúde (CIIS), Faculdade de Medicina, Universidade Católica Portuguesa de Lisboa, 2635-631 Lisbon, Portugal

**Keywords:** chromic myeloid leukemia, genetic variants, antioxidant defenses, NRF2/KEAP1 pathway, overall survival, tyrosine kinase inhibitor resistance

## Abstract

Chronic myeloid leukemia (CML) is a clonal myeloproliferative neoplasia characterized by the *BCR::ABL1* fusion gene, which codifies the BCR-ABL protein with increased tyrosine kinase activity. Despite the clinical results for the outstanding tyrosine kinase inhibitors (TKIs), drug resistance is a problem in CML management. Genetic variants that alter redox homeostasis by changing antioxidant enzyme expression or activity may influence patient responses and could enhance patient stratification. We aimed to assess the association of *SOD2*, *CAT GPX1*, *NRF2*, and *KEAP1* genetic variants with TKI response and disease prognosis. For this purpose, we genotyped the variants rs4880 (*SOD2*), rs1050450 (*GPX1*), rs1001179 (*CAT*), rs6721961, rs4893819, rs35652124, rs6706649, rs13001694 (*NFE2L2*), and rs113540846 (*KEAP1*) via PCR in 187 CML patients. Our results show that variants in genes related to oxidative stress influence the development and degree of TKI resistance (allele G and GG genotypes of *GPX1* and CT genotype of *NFE2L2* rs4893819), the appearance of mutations in the *BCR::ABL1* gene (AG genotype of *NFE2L2* rs13001694 and genetic profile GGCTTCCCGG of the *NFE2L2*/*KEAP1* axis), disease evolution (AG genotype of *SOD2* and CT genotype of *NFE2L2* rs4893819), and overall survival (CC genotype of *CAT* and GG genotype of *NFE2L2* rs13001694) of CML patients. Our study found that variants in oxidative stress-related genes impact treatment response and outcomes in CML.

## 1. Introduction

Chronic myeloid leukemia (CML) is a clonal myeloproliferative neoplasia characterized by the presence of the *BCR::ABL1* fusion gene as a consequence of a reciprocal translocation between chromosomes 9 and 22 [1]. Currently, and based on the constitutive tyrosine kinase activity of BCR-ABL oncoprotein, the therapeutic protocols are centered on tyrosine kinase inhibitors (TKIs) such as imatinib, dasatinib, and nilotinib [2]. Despite the excellent clinical results of TKIs, the emergence of drug resistance has become a problem in managing CML [3]. Multiple molecular mechanisms contribute to TKI resistance, including *BCR::ABL1* point mutations, altered drug transporter activity, DNA repair and genomic instability, activation of alternative signaling pathways, epigenetic dysregulation, and oxidative stress [4].

Oxidative stress (OS) is related to the development and progression of various pathologies, including cancer [5]. This stress state results from the imbalance between reactive oxygen species (ROS) production and the antioxidant defense levels, which neutralize the former molecules [6,7,8]. At the physiological levels, and due to their role as second messengers in intracellular signaling pathways, ROS control cell division, proliferation, and survival [9]. However, long-term ROS exposure induces damage in proteins, lipids, and DNA, contributing to neoplasia development, progression, and drug resistance [10]. In CML, as in other hematological malignancies, the increase in ROS has been described and associated with the carcinogenic process [11]. Particularly in CML, BCR-ABL oncoprotein activity leads to ROS generation, resulting in an oxidative stress environment prone to inducing DNA damage and genomic instability [12]. Multiple drugs, as in the case of TKIs, increase the levels of exogenous ROS, inducing cell death [5]. The nuclear factor erythroid 2-related factor 2 (NRF2)/kelch-like ECH-associated protein 1 (KEAP1) pathway is one of the main regulators of oxidative homeostasis. In the presence of oxidative stress, NRF2 dissociates from KEAP1 and translocates to the nucleus [13]. This transcription factor, codified by the *NFE2L2* gene, induces the expression of genes that play a role in redox homeostasis, DNA repair, drug excretion, survival, and autophagy. The cytoprotective and antioxidant genes, such as *GPX1*, *CAT*, and *SOD2,* are some examples of NRF2-regulated genes that could be implicated in tumor progression, metastasis, and resistance to chemotherapy [14,15,16]. The genetic background of each patient can influence all these intricate signaling pathways associated with cellular redox homeostasis. We hypothesize that germinative genetic variants, such as single nucleotide variants (SNVs), which are associated with altered expression levels and enzymatic activity in OS key player genes, may influence CML development, prognosis, and treatment response.

In this context, and given the relevance of oxidative stress homeostasis in CML treatment and prognosis, we evaluated the possible association of *NFE2L2*, *KEAP1*, *SOD2*, *CAT*, and *GPX1* genetic variants with TKI response (including the response rates, number of required lines of TKI treatment, and presence of *BCR::ABL1* mutations) and disease prognosis (progression and overall survival).

## 2. Results

### 2.1. Characteristics of CML Patients

Our cohort of CML patients included 187 CML patients, composed of 110 (58.8%) males and 77 (41.2%) females with a median age of 54 years (range: 15–86). Most patients were diagnosed in the chronic phase (94.6%, n = 177) (Table 1). Patients were classified as resistant if they required two or more lines of TKI treatment. In this context, 138 (73.8%) patients were categorized as TKI responders, while 49 (26.2%) were classified as TKI-resistant. No statistically significant differences were found between these two sub-groups of patients according to demographic features and the clinical parameters not associated with resistance (Table 1). Nearly all CML patients received imatinib as a first-line TKI (95.2%, n = 178). In the resistant sub-group, all the patients were treated with imatinib up-front. Furthermore, 12 patients (6.4%) were exposed to three or more TKI lines during treatment, and 22 (21.2%) developed *BCR::ABL1* mutations. In this cohort, only 8 patients progressed (4.3%) to advanced forms, with all of them belonging to the TKI-resistant group, and 27 patients (14.4%) died.

### 2.2. Genetic Variants Associated with TKI Response and BCR::ABL1 Mutational Status

The association of selected genetic variants with the response to TKI treatment was evaluated to infer the contribution of these SNVs in the response profile. We performed multiple analyses according to allele and genotype distribution and applied four genetic models described in the Materials and Methods section. To achieve this goal, we correlated the SNVs with TKI response (sensitive and resistant patient subgroups), the number of TKI lines of treatment needed, and the *BCR::ABL1* mutational status.

The allele distribution in TKI-sensitive and TKI-resistant patients is represented in Table 2. Patients carrying allele G of *GPX1* rs1050450 had a higher probability of developing TKI resistance (OR = 1.841, 95%CI = 1.108–3.059, *p* = 0.020). The same association was observed in allele G of *KEAP1* rs113540846, with a probability of becoming resistant to TKI treatment that was 31 times higher (OR = 31.07, 95%CI = 1.886–511.9, *p* < 0.0001) (Table 2).

We observed significant associations between specific genotypes and the response profiles (Table 3). All genotypes among the study groups were in Hardy–Weinberg equilibrium (HWE). For *GPX1* rs1050450, the patients homozygotic for allele G presented a risk of failing the first-line TKI treatment which was two times higher (MD: OR = 2.199, 95%CI = 1.120–4.316, *p* = 0.022) (Table 3). Furthermore, the resistant patients may require more than two lines of treatment, and this could be related to the SNVs observed in the study. We observed that patients heterozygotic to the *NFE2L2* rs4893819 variant had 5.6 times higher risk of requiring three or more lines of treatment (highly resistant profile) during the disease’s course (MOD: OR = 5.600, 95%CI = 1.218–25.751, *p* = 0.027) (Table 3). Moreover, the presence of a *BCR::ABL1* point mutation was the most common resistance mechanism evaluated in the case of lack of response. Over the multiple genetic variants evaluated, *NFE2L2* rs13001694 was associated with the *BCR::ABL1* mutational status. Patients with the genotype AG presented a probability of developing mutations in the fusion gene which was 8.5 times higher (MCD: OR = 8.571, 95%CI = 1.004–73.210, *p* = 0.050; MOD: OR = 12.000, 95%CI = 1.429–100.754, *p* = 0.022) (Table 3). Additionally, the impact of gender was also assessed, and no significant associations were identified.

To assess the impact of the multiple genetic variants on the response profile, we performed haplotype and genotypic profile (GP) analysis using Arlequin software. The haplotype analysis was performed for the *NFE2L2* (rs6721961/rs4893819/rs35652124/rs6706649) genetic variants. The GP analysis was performed and grouped in three categories: (1) the global profile with seven SNVs (*NFE2L2*: rs6721961/rs4893819/rs35652124/rs6706649/*SOD2*: rs4880/*GPX1*: rs1050450/*KEAP1*: rs113540846); (2) the *NFE2L2/KEAP1* axis (*NFE2L2*: rs6721961/rs4893819/rs35652124/rs6706649/*KEAP1*: rs113540846); and (3) antioxidant defenses (*SOD2* rs4880/*GPX1* rs1050450). For all the variants related to TKI response, we did not observe any association with *NFE2L2* haplotypes. Regarding the GP analysis of the *NFE2L2/KEAP1* axis, patients with GP GG CT TC CC GG presented an increased risk of *BCR::ABL1* mutations (OR = 6.000, 95%CI = 1.730–20.810, *p* = 0.006).

### 2.3. Impacts of Studied SNVs on Progression and Overall Survival

The link between the selected genetic variants and prognosis was evaluated by the association with CML progression and evolution and overall survival. In terms of allele distribution, we did not observe any association with disease progression or survival. However, the genotypic analysis revealed an association between *CAT* rs1001179 and the CML patients’ overall survival. The CC genotype showed an increased risk of death (MD: OR = 5.100, 95%CI = 1.125–23.117, *p* = 0.035) while the CT genotype showed a protective effect (MCD: OR = 0.163, 95%CI = 0.027–0.969, *p* = 0.046) (Table 4).

**Figure 1:**
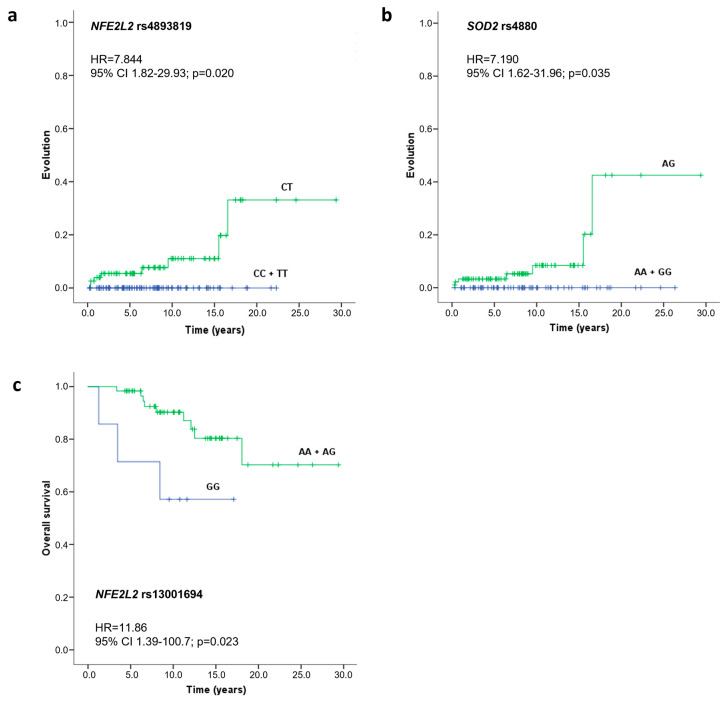
Evolution and overall survival curves of CML patients according to *NFE2L2* rs4893819 (**a**), *SOD2* rs4880 (**b**), and *NFE2L2* rs13001694 (**c**) genotypes. Time to evolution and survival analysis were performed using the Kaplan Meier method, differences in survival were tested with a log-rank test, and the hazard ratio (HR) with a 95% confidence interval (CI) was calculated using the Cox proportional hazard model.

The impact of different SNVs on CML prognosis was also evaluated by estimating the rate of disease progression and overall survival. This analysis was performed using the Kaplan-Meier method, and patients were stratified according to their SNV genotypes. As observed in Figure 1, the *NFE2L2* rs4893819 CT genotype (HR = 7.844, 95%CI = 1.82–29.93, *p* = 0.020) and the *SOD2* rs4880 AG genotype (HR = 7.190, 95%CI = 1.62–31.96, *p* = 0.035) were significantly associated with higher progression rates in comparison with the homozygotic genotypes of *NFE2L2* rs4893819 and *SOD2* rs4880, respectively (Figure 1a,b). Moreover, CML patients carrying the GG genotype for *NFE2L2* rs13001694 had a significantly lower overall survival time (HR = 11.86, 95%CI = 1.39–100.7, *p* = 0.023) compared with those that were allele A carriers (Figure 1c), with an average overall survival time of 11.7 ± 2.5 years compared with 24.3 ± 1.6 years for patients with the AA and AG genotypes.

Haplotype and genotypic profile (GP) analyses of the studied SNVs were performed as previously described, and the contributions to CML prognosis were assessed. According to the *NFE2L2* haplotype analysis, we identified 11 haplotypes in our CML patient’s cohort. Three of them (GCC, GCTC, and TTTT) were identified only in patients who showed disease progression, while GCTC, GTCC, GTCT, GTTT, and TCTT were only observed in the opposite group. The haplotype GTTC was associated with six times higher risk of disease progression (OR = 6.160, 95%CI = 1.338–28.36, *p* = 0.036). Furthermore, the genotypic analysis identified an association between the GP of antioxidant defenses with CML progression. The GP AG GG antioxidant defenses (*SOD2* rs4880/*GPX1* rs1050450) conferred a higher risk of disease progression (OR = 4.615, 95%CI = 1.083–19.67, *p* = 0.047).

### 2.4. Influence of Studied SNVs on Gene Expression Levels and Protein Function

Genotype-tissue expression (GTEx) analysis in whole blood samples was possible for seven of the nine SNVs tested, with missing information for *NFE2L2* rs6706649 and rs13001694. Over the SNVs evaluated, homozygous samples for the polymorphic allele showed significantly lower expression levels than those homozygous for the reference allele. *CAT* rs1001179 and *KEAP1* rs113540846 were the exceptions, as SNVs resulted in higher gene expression levels according to GTEx analysis (Supplementary Figure S1).

For the SNVs that resulted in a missense variant, we used predictive tools to assess the impact on protein function and stability. Regarding protein function, *SOD2* rs4880 and *GPX1* rs1050450 were classified as tolerated effects according to Sort Intolerant from Tolerant (SIFT) and benign by Polymorphism Phenotyping v2 (PolyPhen-2) analysis. Concerning protein stability, the predicted stability change (ΔΔG^Stability^) was −0.23 kcal/mol for *SOD2* rs4880 and −0.56 kal/mol for *GPX1* rs1050450, indicating a destabilizing effect from the variants on the protein structure.

## 3. Discussion

Oxidative stress is a key player in the development and progression of neoplasms but also has a relevant role in the treatment efficacy of these diseases [15]. The genetic variability of each patient influences tumor susceptibility, the progression and survival when neoplasms are already installed, as well as the response to oncological treatments [15,17,18,19,20]. Genetic variants that impact redox homeostasis, by altering the expression levels of antioxidant enzymes or redox regulator transcription factors or altering enzyme activities, may present a relevant role in patients’ responses, and they could contribute to better patient stratification. Based on this, we conducted a study focusing on genetic variants of genes essential for maintaining oxidative stress balance and that have been linked to decreased gene expression or lower protein activity.

Focusing on the NRF2 transcription factor (TF), the genetic variants on the *NFE2L2* gene that codify this TF have been described as associated with the development and progression of various solid and hematological cancers [19,21]. However, the link between TKI response in CML patients and these variants has still not been explored. In our study, we observed an association of the *NFE2L2* rs4893819 CT genotype with a higher TKI-resistant phenotype, since the patients carrying this genotype had a 5.6 times higher probability of needing three or more lines of treatment. In accordance with this, the same genotype was correlated with a higher risk of disease evolution. In the literature, the studies evaluating this SNV are scarce, and according to Nunes Dos Santos et al. (2019), this SNV did not present any association with another disease as alcoholic hepatitis [22]. Another *NFE2L2* variant, rs13001694, has been associated with an increased risk of developing myelodysplastic neoplasia and breast, colon, and stomach cancer [23,24]. According to Gonçalves et al. (2017), individuals carrying allele G are more likely to develop myelodysplastic neoplasia when compared with AA carriers [19]. In the context of TKI response, we found that CML patients with AG genotypes for this variant have a higher risk of *BCR::ABL1* mutations, which are highly associated with TKI resistance. On the other hand, the GG genotype gives CML patients treated with TKIs a shorter average life expectancy. Despite the other *NFE2L2* variants studied (rs6721961, rs6706649, and rs35652124) being linked with neoplasia development and other pathologies [25,26,27,28], in our study, we did not find any correlation between them and CML patients’ responses and prognoses. More comprehensively, we also studied the contributions of the different selected SNVs on the *NFE2L2* gene to CML patient’s characteristics via haplotype analysis. In our CML cohort, the *NFE2L2* haplotype GTTC (allele G of SNV rs6721961, T of rs4893819, T of rs35652124, and C of rs6706649) was associated with six times higher risk of disease progression. A similar analysis was performed by Arisawa et al. (2008), and they found that the TC haplotype (*NFE2L2* rs35652124 and rs6706649) was associated with higher levels of inflammation and a greater likelihood of developing gastric mucosal atrophy [29]. Corroborating our results, the GTC haplotype (*NFE2L2* rs6721961, rs35652124, and rs6706649) has been described as having low promoter activity and consequently lower gene expression, which could explain the impact on disease evolution [30].

Germline and somatic variants in the *KEAP1* gene result in reduced activity for the KEAP1 protein, leading to accumulation of the NRF2 transcription factor and, consequently, resistance to chemotherapy [31]. Despite these relevant roles, the SNVs in this gene have not been explored in the context of hematological neoplasms. Studies on the *KEAP1* rs113540846 variant are not described in the literature. However, we found a strong correlation between allele G and an increased risk of developing resistance to TKIs, and GTEx analysis indicated that this allele correlated with lower gene expression levels. Further analysis is needed to explore the relevance and impact of *KEAP1* SNVs in the CML scenario.

Genetic variants of antioxidant enzymes, namely in SOD2, GPX1, and CAT, were already associated with the development of various neoplasms and treatment efficacy [10,32]. For *SOD2*, allele G in the rs4880 variant has been associated with lower gene expression and lower stability of mRNA, which affects the entry of SOD2 into mitochondria. As a result, the antioxidant activity of SOD2 decreases by 30–40%, thus reducing the neutralizing capacity of superoxide anions [33,34]. This particular SNV was associated with the development of chronic kidney disease, breast cancer, lung cancer, and acute myeloid leukemia, among other pathologies [28,32]. According to Xu et al. (2015), the AG and GG genotypes have a negative impact on survival for Chinese gastric cancer patients who receive platinum- and fluorouracil-based adjuvant chemotherapy [35]. Along the same line, the G carrier genotypes of this variant were associated with higher stages of disease in papillary thyroid carcinoma [36]. Moreover, the *SOD2* rs4880 AG genotype was associated with non-chemotherapy response in breast cancer patients [37]. However, the contribution to CML response and prognosis is poorly explored. In accordance, in our study, we observed a correlation of the AG genotype with disease progression, where AG patients progressed seven times faster compared with those with the AA and GG genotypes. A crucial antioxidant defense to detoxification of hydrogen peroxide is glutathione peroxidase 1, and genetic variants on *GPX1* have been linked with neoplasia development [38,39,40]. In particular, the rs1050450 variant, which consists of a substitution amino acid change from proline to leucine, causes a decline in GPX1 activity due to a conformational change in the protein [41,42]. According to Kagita et al. (2021), the GA and GG genotypes of *GPX1* rs1050450 were identified as being a risk factor of CML development [43]. Furthermore, in the same study, patients homozygous to allele A of the rs1050450 variant, which encodes an enzyme with reduced activity [38,43], were associated with a less profound molecular response and the development of *BCR::ABL1* mutations. In contrast, in our CML cohort, the allele G and GG genotypes were associated with a worse response to TKIs, and these patients were more likely to become TKI-resistant. In accordance with our results, the GG genotype was associated with a higher risk of breast cancer and with higher detoxification activity due to higher GPX1 activity in these individuals [41,44].

Another relevant enzyme in the detoxification of hydrogen peroxide is catalase, which is encoded by the *CAT* gene. For this gene, several genetic variants have been studied, with *CAT* rs1001179 being the one known in more detail. This variant occurs in the promoter region and affects the association of transcription factors to the promoter, leading to changes in the transcription rate [45,46]. As a consequence, individuals carrying TT genotypes present lower CAT activity in comparison with CC genotype carriers [47,48]. Due to its important role in redox homeostasis, this variant has been identified as a risk factor for CML, hepatocellular carcinoma, breast cancer, and gastric cancer development [43,49,50,51]. However, the reports with this variant are not consensual, and some authors failed to identify this risk association, particularly for CML [52]. In our study, the presence of the CC genotype was associated with an increased probability of death, while the opposite effect was observed for the CT genotype. According to our results, the presence of allele T was associated with a higher probability of survival. In accordance with the findings previously described by Kagita et al. (2021) [43], we did not find any association between this *CAT* SNV and the *BCR::ABL1* mutational status.

For a more comprehensive and integrated investigation of the impact of several SNVs at the same time, we performed a genotypic profile (GP) analysis to identify profiles that could contribute to a better prediction of CML TKI response and prognosis. The GP of the *NFE2L2*/*KEAP1* axis, GG CT TC CC GG, was related to a higher risk of *BCR::ABL1* mutations, a crucial event for TKI efficacy, while the antioxidant defense GP (SOD2 rs4880/GPX1 rs1050450) AG GG was associated with a high risk of disease progression. In previous work from our group in the same patient cohort, we identified genetic profiles in the ABC and SLC drug transporter genes related to TKI response and the degree of resistance [20]. However, a similar analysis with oxidative stress-related genes was not described in the CML field.

The studies of genetic variants and their link with neoplasia prognosis and treatment response have been commonly associated with conflicting results. These heterogeneous results can be explained by limitations in sample size, SNV ethnic differences, treatment, and response criteria [53]. Furthermore, epigenetic mechanisms, such as DNA methylation or post-translational modifications of histones, can also influence gene transcription, altering expression regardless of the germline genetic variant present [54]. In particular, we recognize that the sample size may constitute a limitation in our work, and further analysis could be performed for a more complete view of the selected SNVs’ contribution to CML treatment response and prognosis. In this line, future analyses could complement the performed functional studies with predictive tools, particularly to assess gene expression and protein activity in a patient’s cohort. Still, the identification of relevant SNV profiles can significantly impact therapeutic monitoring and improve TKI response, namely by improving the mutation screening in patients with higher risk of *BCR::ABL1* mutations or switching to a different TKI in cases of patients at high risk of needing multiple lines of treatment. The inclusion of SNV analysis can increase patient quality of life and consequently reduce the burden on the healthcare system.

## 4. Materials and Methods

### 4.1. Study Population

In this retrospective study, we enrolled 187 CML patients recruited from three hospitals—Centro Hospitalar e Universitário de Coimbra (ULSC/CHUC), Hospital Distrital da Figueira da Foz (HDFF), and Instituto Português de Oncologia de Lisboa (IPO-Lisboa)—between September 2014 and August 2017. Patients were diagnosed based on the World Health Organization (WHO) classification, and treatment response criteria were defined according to European Leukemia Net (ELN) guidelines [2]. Blood samples were obtained during monitoring consultations conducted by the clinical team.

The characteristics of the CML patients included in this study are summarized in Table 1. The patient group was subdivided into responsive (n = 138) or resistant (n = 49) to TKIs, based on the therapeutic outcomes for prognostic analysis (Table 1). To avoid confounding effects, the patients intolerant to therapy were excluded. This study was conducted according to the Helsinki Declaration, and all participants provided written informed consent for participation before enrolment. All research procedures were approved by the Faculty of Medicine Ethics Committee (University of Coimbra, Portugal) (ref. CE-014/2014).

### 4.2. Gene and SNV Selection

The genes to be studied were selected based on their relationship with oxidative stress. According to the literature, the *NFE2L2* and *KEAP1* genes encode the NRF2 and KEAP1 proteins, respectively, and are crucial players in redox homeostasis. The *CAT*, *GPX1*, and *SOD2* genes encode the antioxidant proteins catalase, glutathione peroxidase 1, and manganese superoxide dismutase, respectively. The genetic variants of these genes were selected based on the frequency of the minor allele (MAF), which had to be greater than 10% in the European population (ALFA allele frequency). After this screening, the variants selected were rs6721961, rs4893819, rs35652124, rs6706649, and rs13001694 from the *NFE2L2* gene, rs113540846 from the *KEAP1* gene, rs4880 from the *SOD2* gene, rs1050450 from the *GPX1* gene, and rs1001179 from the *CAT* gene. In the Supplementary Materials (Table S1), the characterization of the selected SNVs is detailed.

### 4.3. Genotyping

Blood samples were collected in EDTA tubes, and NZYol reagent (NZYtech, Lisbon, Portugal) was used to extract genomic DNA from the whole blood according to the manufacturer’s protocol. The DNA quality and quantity were determined using a NanoDrop ND-1000 spectrophotometer (NanoDrop Technologies, Wilmington, NC, USA). For each genotyping reaction, 100 ng of DNA was used, and SNVs were genotyped through tetra-primer ARMS-PCR, ASO-PCR, and RFLP-PCR assays. The primers for the different reaction were designed using BatchPrimer3, version 1.0 (USDA-ARS, Albany, CA, USA) (http://probes.pw.usda.gov/batchprimer3/ (accessed on 5 October 2020)) [55]. The primers and PCR reaction conditions are described in the Supplementary Materials (Table S2). The results were initially confirmed via direct sequencing. Samples discovered to contain the three potential genotypes were employed as positive controls in each genotype assay. Genotyping was repeated in approximately 10% of the total samples as a quality check to ensure accuracy.

### 4.4. Genetic Analysis

In this work, we started by analyzing the allele and genotype distribution. In this analysis, it was important to understand the concepts of minor (m) and major (M) alleles. In a biallelic SNV, the minor allele represents the less frequent allele in the population, while the major (M) allele is the most frequent allele and serves as a reference [56]. Different genetic models can be applied to genotypic analysis, including the codominant model (MCD), dominant model (MD), recessive model (MR), and overdominant model (MOD). Each of these models represents different assumptions about genetic effects. The codominant model (MCD), where each genotype was compared with the homozygous of a major allele (mm or mM vs. MM), assumes each genotype individually and determines the risk associated with it compared to the reference genotype. The dominant model, in which minor allele carriers are analyzed against the major allele homozygous (mm + mM vs. MM), assumes that having one or more copies of the major allele increases risk. When minor allele homozygotes are compared to major allele carriers (mm vs. MM + mM), this corresponds to the recessive model (MR), which assumes the need for two copies of a minor allele to alter the risk associated with that variant. Lastly, the overdominant model (MOD), where heterozygous individuals are compared to homozygous individuals (mM vs. MM + mm), explores the combination of both alleles to confer risk [57,58].

### 4.5. Statistical Analysis

The statistical analysis was conducted in collaboration with the Laboratory of Biostatistics and Medical Informatics at the Faculty of Medicine of the University of Coimbra (FMUC). The frequencies of alleles and genotypes were determined by direct counting. The frequencies of the genotypic profile (GP) and the Hardy–Weinberg equilibrium (HWE) in the studied groups were determined using Arlequin software version 3.5.1.2 (CMPG, University of Bern, Switzerland) [59]. The comparison of allele frequencies was conducted using Fisher’s exact test in GraphPad Prism, version 9.0 (GraphPad Software, San Diego, CA, USA). The associations between genotype, haplotype, GP, and clinical characteristics were analyzed using odds ratio (OR) and 95% confidence interval (CI) calculations. This was performed through unconditioned logistic regression in SPSS, version 29.0 (IBM, New York, NY, USA). We used the Kaplan–Meier method in SPSS to estimate the time to disease progression and overall survival of patients grouped based on their genotypes. We tested for differences using the log-rank statistic. The hazard ratio (HR) and its 95% confidence interval (CI) were calculated using the Cox proportional hazard model. All statistical analyses were two-sided, and we considered *p* < 0.05 statistically significant.

GTEx portal v10 (https://www.gtexportal.org/home/) was accessed on 10 April 2025 to evaluate the effect of the selected SNPs on gene expression in whole blood samples. The SIFT algorithm (https://sift.bii.a-star.edu.sg/) and PolyPhen-2 (http://genetics.bwh.harvard.edu/pph2/) were also accessed on 10 April 2025 to estimate the functional impact of the SNP and the associated amino acid substitution on protein function [60]. The effect of the selected SNPs on protein stability was evaluated using DynaMut online tools (https://biosig.lab.uq.edu.au/dynamut2/), accessed on 10 April 2025.

## 5. Conclusions

In conclusion, the results obtained show that variants in genes related to oxidative stress influence the clinical characteristics of CML patients, namely the development and degree of TKI resistance, mutations in the *BCR::ABL1* gene, the evolution of the disease, and the overall survival of CML patients. Determining these SNVs and combining them with other known variants could improve the prognostic characterization of CML patients and enable a better understanding of inter-individual differences, thus allowing for a more personalized and informed therapeutic choice and contributing to precision medicine.

## Figures and Tables

**Table 1:** Characteristics of CML patients.

Characteristics	CML						
	All Patients (n = 187)		TKI Responders (n = 138)		TKI Resistors (n = 49)		*p* Value
**Demographic Features**							
Gender (%)							
Male	110	(58.8)	78	(56.5)	32	(65.3)	*p* = 0.294 *
Female	77	(41.2)	60	(43.5)	17	(34.7)	
Age (years)							
Median	54		54		50		*p* = 0.408 ^#^
Range	15–86		15–86		18–79		
**Clinical Features**							
Phase of Disease							
Chronic Phase (%)	177	(94.6)	131	(94.9)	46	(93.9)	*p* = 0.091 *
Accelerate Phase (%)	5	(2.7)	5	(3.6)	–	–	
Blast Crisis (%)	5	(2.7)	2	(1.5)	3	(6.1)	
Scoring Systems							
Sokal Score	(n = 138)		(n = 105)		(n = 33)		
Low Risk (%)	75	(54.4)	59	(56.2)	16	(48.5)	*p* = 0.712 *
Intermediate Risk (%)	45	(32.6)	33	(31.4)	12	(36.4)	
High Risk (%)	18	(13.0)	13	(12.4)	5	(15.1)	
Euro Score	(n = 138)		(n = 105)		(n = 33)		
Low Risk (%)	100	(72.5)	79	(75.2)	21	(63.7)	*p* = 0.266 *
Intermediate Risk (%)	32	(23.2)	21	(20.0)	11	(33.3)	
High Risk (%)	6	(4.3)	5	(4.8)	1	(3.0)	
EUTOS Score	(n = 136)		(n = 104)		(n = 32)		
Low Risk (%)	119	(87.5)	92	(88.5)	27	(84.4)	*p* = 0.528 *
High Risk (%)	17	(12.5)	12	(11.5)	5	(15.6)	
First-Line TKI							
Imatinib (%)	178	(95.2)	129	(93.5)	49	(100.0)	*p* = 0.700 *
Other TKI (%)	9	(4.8)	9	(6.5)	–	–	
Number of TKIs during Treatment							
1 TKI (%)	138	(73.8)	138	(100.0)	–	–	*p* < 0.001 *
2 TKIs (%)	37	(19.8)	–	–	37	(75.5)	
≥3 TKIs (%)	12	(6.4)	–	–	12	(24.5)	
Mutations on *BCR::ABL1*	(n = 104)		(n = 69)		(n = 35)		
Present (%)	22	(21.2)	11	(15.9)	11	(31.4)	*p* = 0.068 *
Absence (%)	82	(78.8)	58	(84.1)	24	(68.6)	
Disease Evolution							
Progression (%)	8	(4.3)	–	–	8	(16.3)	*p* < 0.001 *
No Progression (%)	179	(95.7)	138	(100.0)	41	(83.7)	
Overall Survival	(n = 194)		(n = 138)		(n = 49)		
Death (%)	27	(14.4)	12	(8.7)	15	(30.6)	*p* < 0.001 *
Survive (%)	160	(85.6)	126	(91.3)	34	(69.4)	

* Chi-square test analysis between TKI responder and TKI-resistant groups. # Mann–Whitney test analysis between TKI responder and TKI-resistant groups. Bold indicates a statistically significant association.

**Table 2:** Allele distribution based on TKI response.

	*GPX1* rs1050450		*KEAP1* rs113540846	
	Allele G	Allele A *	Allele G	Allele A *
TKI-sensitive	155	115	190	44
TKI-resisitant	67	27	66	0
OR (95% CI)	**1.841 (1.108–3.059)**		**31.07 (1.886–511.9)**	
*p* value	**0.020**		**<0.0001**	

Bold indicates a statistically significant association obtained by Fisher’s exact test, and * indicates the reference allele. OR = odds ratio; CI = confidence interval.

**Table 3:** The distribution of significant genotypes of the selected SNVs in CML patients according to TKI response profile.

Gene: dbSNV		n	%	OR (95% CI)		*p* Value	n	%
***GPX1* rs1050450**		**TKI-resistant**					**TKI-sensitive**	
GG		25	53.2	Ref.			46	34.1
GA		17	36.2	0.497 (0.241–1.024)		0.058	63	46.7
AA		5	10.6	0.354 (0.121–1.036)		0.058	26	19.3
GG (MD)				**2.199 (1.120–4.316)**		**0.022**		
AA (MR)				0.499 (0.180–1.386)		0.182		
GA (MOD)				0.648 (0.327–1.284)		0.213		
***NFE2L2* rs4893819**		**3 or more TKIs**					**2 TKIs**	
CC		3	37.3	Ref.			11	35.5
CT		8	72.7	2.933 (0.605–14.231)		0.182	10	32.3
TT		0	0.0	-		-	10	32.3
CC (MD)				0.682 (0.150–3.109)		0.621		
TT (MR)				-		-		
CT (MOD)				**5.600 (1.218–25.751)**		**0.027**		
***NFE2L2* rs13001694**		**With mutation**					**Without mutation**	
AA		1	7.7	Ref.			15	35.7
AG		12	92.3	**8.571 (1.004–73.210)**		**0.050**	21	50
GG		0	0.0	-		-	6	14.3
AA (MD)				0.150 (0.018–1.269)		0.082		
GG (MR)				-		-		
AG (MOD)				**12.000 (1.429–100.754)**		**0.022**		

The OR (95% CI) and *p* values were obtained by unconditioned logistic regression according to the four genetic models. Bold indicates a statistically significant association. CI = confidence interval; MD = dominant model; MOD = overdominant model; MR = recessive model; OR = odds ratio; Ref. = reference.

**Table 4:** Significant genotype distribution of *CAT* rs1001179 according to overall survival.

Gene: dbSNV		n	%	OR (95% CI)		*p*-Value	n	%
***CAT* rs1001179**		**Death**					**Survival**	
CC		4	33.3	Ref.			5	8.9
CT		3	25.0	**0.163 (0.027–0.969)**		**0.046**	23	41.1
TT		5	41.7	0.223 (0.044–1.131)		0.07	28	50.0
CC (MD)				**5.100 (1.125–23.117)**		**0.035**		
TT (MR)				0.714 (0.202–2.522)		0.601		
CT (MOD)				0.478 (0.117–1.961)		0.306		

The OR (95% CI) and *p* values were obtained with unconditioned logistic regression according to the four genetic models. Bold indicates a statistically significant association. CI = confidence interval; MD = dominant model; MOD = overdominant model; MR = recessive model; OR = odds ratio; Ref. = reference.

## Data Availability

The original contributions presented in this study are included in the article or Supplementary Materials. Further inquiries can be directed to the corresponding author.

## Supplementary Materials

The following supporting information can be downloaded at https://www.mdpi.com/article/10.3390/ijms26125682/s1.

## Abbreviations

The following abbreviations are used in this manuscript:

ASO-PCR	Allele-specific oligonucleotide polymerase chain reaction
CAT	Catalase
CI	Confidence interval
CML	Chronic myeloid leukemia
ELN	European Leukemia Net
GP	Genotypic profile
GPX1	Glutathione peroxidase 1
HR	Hazard ratio
HWE	Hardy–Weinberg equilibrium
KEAP1	Kelch-like ECH-associated protein 1
M	Major allele
m	Minor allele
MAF	Frequency of the minor allele
MCD	Codominant model
MD	Dominant model
MgCL2	Magnesium chloride
MOD	Overdominant model
MR	Recessive model
NFE2L2	Nuclear factor erythroid 2-related factor 2 gene
NRF2	Nuclear factor erythroid 2-related factor 2
OD	Odds ratio
OS	Oxidative stress
RFLP-PCR	Restriction fragment length polymorphism polymerase chain reaction
Ref	Reference allele or genotype
ROS	Reactive oxygen species
SNV	Single nucleotide variants
SOD2	Superoxide dismutase [Mn], mitochondrial
Ta	Annealing temperature
TETRA-ARMS-PCR	Tetra-primer amplification refractory mutation system-polymerase chain reaction
TF	Transcription factor
TKI	Tyrosine kinase inhibitor
WHO	World Health Organization
