# Supplementary material for: Genetic Variants in Oxidative Stress-Related Genes and Their Impact on Prognosis and Treatment Response in Chronic Myeloid Leukemia Patients
Source: Int J Mol Sci. 2025 Jun 13;26(12):5682. doi: 10.3390/ijms26125682 (PMC12193163; doi:10.3390/ijms26125682)
Supplement: Supplementary file 1 [file ijms-26-05682-s001.zip › ijms-3618159-supplementary.pdf]

Supplementary Material

Genetic variants in oxidative stress-related genes and their impact on prognosis and treatment response in chronic myeloid leukemia patients

Raquel Alves [1,2,3], Filipa Ventura[1], Joana Jorge [1,2,3], Gilberto Marques [4], Margarida Coucelo [2,5], Joana Diamond [6], Bárbara Oliveiros [2,7], Amélia Pereira [2,8,9], Paulo Freitas-Tavares [5], António M. Almeida [10,11], Ana Cristina Gonçalves [1,2,3*] and Ana Bela Sarmento-Ribeiro [1,2,3,5,]

Table S1. Characterization of the 9 selected SNVs

Gene Symbol*	Protein	dbSNP	Chr. Position†	Functional Consequence	European MAF‡	Clinical significance$
NFE2L2	NRF2	rs6721961	2:177265309	2KB Upstream Variant	0.11612 (T)	Not Reported
		rs4893819	2:177266406	2KB Upstream Variant	0.47480 (C)	Not Reported
		rs6706649	2:177265343	2KB Upstream Variant	0.88200 (C)	Not Reported
		rs35652124	2:177265345	2KB Upstream Variant	0.67283 (T)	Not Reported
		rs13001694	2:177254262	Intron Variant	0.62720 (A)	Not Reported
KEAP1	KEAP1	rs113540846	19:10505230	2KB Upstream Variant	0.82191 (G)	Not Reported
SOD2	SOD2	rs4880	6:159692840	Missense Variant	0.50190 (A)	Benign<br>Likely risk allele
CAT	CAT	rs1001179	11:34438684	2KB Upstream Variant	0.77827(C)	Uncertain significance
GPX1	GPX1	rs1050450	3:49357401	Missense Variant	0.67340 (G)	Benign

*According to HUGO Gene Nomenclature Committee (HGNC). †Chromosome position based on GRCh38.p12. ‡MAF, minor allele frequency according to the ALFA Allele Frequency (European population). $Clinical significance reported on ClinVar database. *NFE2L2*: nuclear factor erythroid 2-related factor 2; *KEAP1:* kelch-like ECH-associated protein 1; *SOD2:* superoxide dismutase [Mn], mitochondrial; *CAT:* catalase; *GPX1*: glutathione peroxidase 1.

Table S2. Genotyping general conditions

Gene	dbSNP	Primers (5′→ 3′)	PCR	PCR Conditions	PCR Products
NFE2L2	rs6721961	FI: GGGCCCTGCCTAGGGGAGATGTGGACAACG<br>RI: TCAGGGTGACTGCGAACACGAGCTGCCAGA<br>FO: CACTTTACCGCCCGAGAATGGCGCCAGC<br>RO: CGTGGTGGCTGCGCTTTGGTGGGAAGAG	TETRA-ARMS-PCR	Ta 60 °C<br>$MgCl_2$ 2.5 mM	Control: 344 bp<br>Allele G: 228 bp<br>Allele T: 176 bp
	rs4893819	FI: TTAACAATTCAAGTTACTTATTAAAATGAC<br>RI: CTCATTGTCTACCTTCTCTGATGGCA<br>FO: AATTACTTGTAATTGAAGCAAGCTTCTT<br>RO: GAAAAGTGAAGGTTATTTCATTCAGTCT	TETRA-ARMS-PCR	Ta 55 °C<br>$MgCl_2$ 2.5 mM	Control: 386 bp<br>Allele T: 251 bp<br>Allele C: 191 bp
	rs6706649	FI: GCTCGTGTTCGCAGTCACCCTGAACTCC<br>RI: ATGGAGACACGTGGGAGTTCAGAGGCGA<br>FO: CGCCTCCCTGATTTGGAGTTGCAGAACC<br>RO: GTGGGAAGAGGTTCTCTTGGGGTTCCCG	TETRA-ARMS-PCR	Ta 58 °C<br>$MgCl_2$ 2.0 mM	Control: 371 bp<br>Allele T: 253 bp<br>Allele C: 174 bp
	rs35652124	FI: GTGTTCGCAGTCACCCTGAACGCACT<br>RI: GAGAATGGAGACACGTGGGAGTTCAGATGG<br>FO: TGATTTGGAGTTGCAGAACCTTGCCCTG<br>RO: CTGCGCTTTGGTGGGAAGAGGTTCTCTT	TETRA-ARMS-PCR	Ta 60 °C<br>$MgCl_2$ 2.5 mM	Control: 373 bp<br>Allele C: 249 bp<br>Allele T: 180 bp
	rs13001694	FO: TTTAGTTTATCCTTTTGTTACCAATTCC<br>RO: GAAATGGCAGAATATTACTTTCTTGTTT<br>FI: GATCTGGACAAGTCACTCTACCTTCA<br>RI: AAGGTAGAGTCTGGGTATATTTTATTGAC	TETRA-ARMS-PCR	Ta 60 °C<br>$MgCl_2$ 2.5 mM	Control: 294 bp<br>Allele A: 191 bp<br>Allele G: 157 bp
KEAP1	rs113540846	FA: AGATCGCGCCACCACACTCCAGCATA<br>RA: TCTCGAACTCCTAACCTCAGGTGATCCA<br>FG: TAAAGAATTCATCTAGGCTGGGTGCGG<br>RG: TTTTTGAGACAGAGTCTCACTCTGTTGAAC	ASO-PCR	Ta 61 °C<br>$MgCl_2$ 2.0 mM	Allele G: 300 bp<br>Allele A: 214 bp
SOD2	rs4880	F: TTCCTCGGCAGCCCAGCCTGCGTAGAC<br>R: TGACGTTCAGGTTGTTCACGTAGGCCGCG	RFLP-PCR	[24]	242 bp
CAT	rs1001179	F: GCTGCTCGGCGCTAGGCAGGCCAAGA<br>R: GCGCAAGGCCCCACCCAGCAGG	RFLP-PCR	[24]	291 bp
GPX1	rs1050450	F: TGTGCCCCTACGCAGGTACA<br>R: CCCCCGAGACAGCAGCA	RFLP-PCR	[24]	141 bp

$MgCl_2$: Magnesium chloride; Ta: Annealing temperature

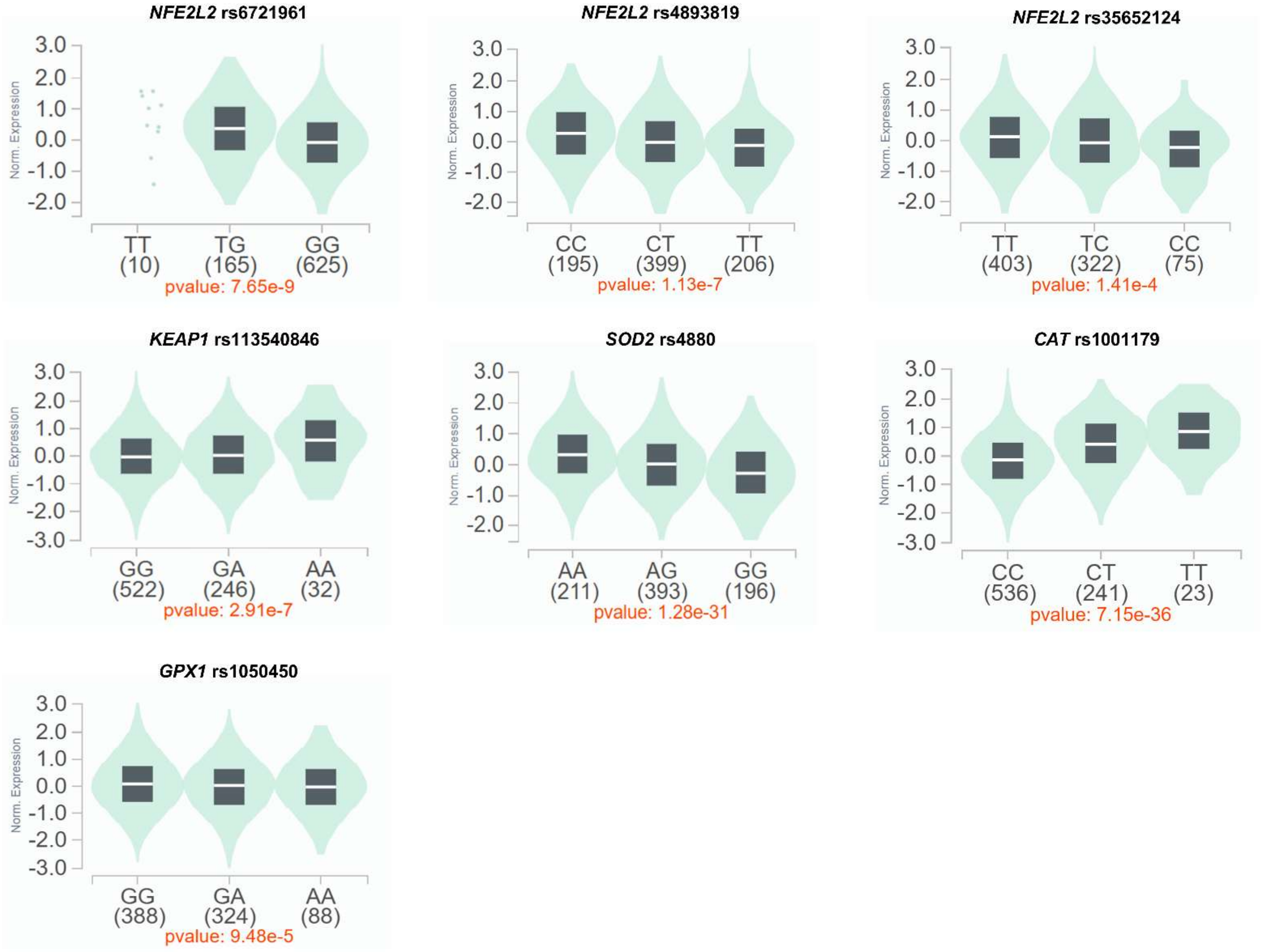

Figure S1. The impact of the studied SNPs on gene expression in whole blood samples from GTEx analysis. Data for *NFE2L2* rs6706649 and rs13001694 were not available in whole blood samples.
